# Myeloid Cell Crosstalk Regulates the Efficacy of the DNA/ALVAC/gp120 HIV Vaccine Candidate

**DOI:** 10.3389/fimmu.2019.01072

**Published:** 2019-05-14

**Authors:** Monica Vaccari, Slim Fourati, Dallas R. Brown, Isabela Silva de Castro, Massimiliano Bissa, Luca Schifanella, Melvin N. Doster, Kathryn E. Foulds, Mario Roederer, Richard A. Koup, Yongjun Sui, Jay A. Berzofsky, Rafick-Pierre Sekaly, Genoveffa Franchini

**Affiliations:** ^1^Animal Models and Retroviral Vaccines Section, Vaccine Branch, National Cancer Institute, National Institutes of Health, Bethesda, MD, United States; ^2^Department of Pathology, Case Western Reserve University, Cleveland, OH, United States; ^3^Vaccine Research Center, National Institute of Allergy and Infectious Diseases, National Institutes of Health, Bethesda, MD, United States; ^4^Vaccine Branch, National Cancer Institute, National Institutes of Health, Bethesda, MD, United States

**Keywords:** MDSC, trained immunity, myeloid cells, HIV/SIV, vaccine

## Abstract

Vaccination with DNA-SIV + ALVAC-SIV + gp120 alum results in inflammasome activation, high levels of IL-1β production, emergency myelopoiesis, and the egress of CXCR4^+^ CD14^+^ pre-monocytes from bone marrow. Previously we have shown that this vaccine-induced innate monocyte memory is associated with decreased risk of SIV_mac251_ acquisition. Because IL-1β also promotes the propagation of monocyte-derived suppressor (M-MDSC)-like cells, here we extended our analysis to this negative regulator subset, characterizing its levels and functions in macaques. Interestingly, we found that DNA prime engages M-MDSC-like cells and their levels are positively associated with the frequency of CD14^+^ classical monocytes, and negatively with the levels of CD16^+^ monocytes, correlates of decreased and increased risk of SIV acquisition, respectively. Accordingly, M-MDSC frequency, arginase activity, and NO were all associated with decrease of CD8 T cells responses and worse vaccination outcome. DNA vaccination thus induces innate immunity by engaging three subsets of myeloid cells, M-MDSCs, CD14^+^ innate monocyte memory, and CD16^+^ monocytes all playing different role in protection. The full characterization of the immunological space created by myeloid cell crosstalk will likely provide clues to improve the efficacy of HIV vaccine candidates.

## Introduction

Immature myeloid cells with a potent inhibitory effect on immunity, including granulocytes, macrophages, and dendritic cells, have been described in humans, macaques, and mice. In recent years, myeloid-derived suppressor cells (MDSCs) have emerged as a major immunosuppressive non-lymphoid population, often linked to immune evasion and unfavorable disease outcome in tumors and infections including HIV (1, 2).

MDSCs are a highly heterogeneous population that includes cells that are morphologically and phenotypically similar to monocytes (monocytic M-MDSCs) and neutrophils (polymorphonuclear PMN-MDSCs) (3). While the nomenclature and phenotypes used to categorize these cell populations vary, human MDSCs are generally defined as cells negative for the expression of MHC class-II HLA-DR and positive for CD33 and CD11b expression. The CD14 or CD15 phenotypic markers are, respectively, used to differentiate between MDSCs derived from monocytes or neutrophils (4).

MDSCs regulate the homeostasis of inflammatory processes (5) and accumulate during unresolved inflammation (6). It is currently unknown whether MDSCs are immature myeloid precursors whose differentiation is blocked during emergency myelopoiesis, or if they are the product of monocyte and neutrophil reprogramming following TLR-signaling and cytokine stimulation (7). The induction of MDSCs is thought to require a combination of long-lasting antigen presentation and strong signals such as growth factors GM-CSF, G-CSF, and other cytokines including IFN-γ, IL-1β, IL-4, IL-6, IL-13, and TNF-α (8–11). The best-known transcription factor regulating MDSC expansion and activity is the signal transducer and activator of transcription 3 (*STAT3*). *STAT3* promotes MDSC survival and blocks their differentiation into mature myeloid cells (12, 13).

MDSCs use a variety of immunosuppressive mechanisms in which the metabolism of the conditionally essential amino acid L-arginine (L-arg) plays a central role. L-arginine can be metabolized by arginase (ARG1 and ARG2), which expression is controlled by *STAT3* (14), and by nitric-oxide synthase 2 (NOS2/iNOS). Both ARG and NOS compete for L-arginine and generate either urea, or citrulline and nitric oxide (NO), respectively (15). In turn, the depletion of extracellular L-arginine and urea production affect the function of the CD3 TCR zeta chain (16). Nitric oxide is one of the most versatile components of the immune system, and numerous immune cells produce and respond to NO (17). NO increases MDSC recruitment in inflammatory sites, inhibits cell proliferation by nitrosylation of receptors, promotes T cell death, and, in the presence of IL-1β, IL-6, IL-23, and TGF-β, favors the development of CD4^+^ T helper producing IL-17 (Th17) and T regulatory cells (Tregs) (18, 19). In addition, MDSCs mediate immunosuppression through reactive oxygen species (ROS), and other mediators such as IL-4 receptor-α (IL-4Rα), programmed death-ligand 1 (PD-L1), interleukin-10 (IL-10), tumor growth factor-β (TGF-β), and phosphorylated *STAT3* (14, 20). While the role of MDSCs in the modulation of T cell responses has been extensively studied, their role in B cell suppression remains poorly understood. Studies have shown MDSCs to both directly regulate B lymphopoiesis (21) and indirectly modulate B cells by generating B regulatory cells (Bregs) (22).

During viral infections, MDSCs or MDSC-like cells suppress CD4^+^ and CD8^+^ T cells proliferation, migration, and function. In addition, a few reports have also described the ability of M-MDSCs to suppress B cell responses (23). MDSCs act as a double-edged sword in HIV/SIV infection (24, 25) by suppressing anti-viral specific immune responses (1, 26), while also antagonizing immune activation (27–29). *Ex vivo* MDSCs derived from HIV-infected patient blood inhibited polyclonal and antigen-specific CD4^+^ and CD8^+^ T cell proliferation and IFN-γ production, but increased FoxP3^+^ CD4^+^ Treg differentiation (18). Interestingly, stimulation of PBMCs with the purified HIV envelope glycoprotein 120 (gp120) *in vitro* induced functional MDSCs capable of suppressing T-cell proliferation (30).

Less is known of the role that vaccination plays in inducing MDSCs, or what effect these cells have on protection. Two recent studies in macaques have shown that MDSCs are induced by influenza and HIV vaccines. Indeed, an mRNA vaccine encoding for influenza hemagglutinin administered in macaques induced both suppressive M-MDSCs (HLA-DR^−^ CD14^+^ cells) and non-suppressive myeloid cells in blood and at the injection site (31). Moreover, a peptide-prime/modified vaccinia Ankara (MVA) boost vaccine regimen induced MDSC-like cells (CD33^+^ CD11b^+^ CD14^+^ DR^low^ cells) and was associated with set-point viral load, suggesting a negative role for M-MDSCs in protection against high viral replication (26).

We previously demonstrated that innate monocyte memory mediated by classical monocytes (HLA-DR^+^ CD14^+^ CD16^−^ cells) is central to the protection elicited by a DNA-SIV + ALVAC-SIV + gp120 alum vaccine administered in macaques (32). While the levels of vaccine-induced classical monocytes and *NLRP3* inflammasome activation were correlated with reduced risk of SIV_mac251_ acquisition (protective), CD16^+^ monocytes and *STAT3* were correlates of increased risk of SIV acquisition (harmful). Given that *STAT3* and IL-1β all result in MDSC accumulation, we studied the kinetics and function of this immunosuppressive subset and its role in protection in macaques vaccinated with the DNA-prime + ALVAC + gp120 boost strategy. Due to the considerable diversity of phenotypic markers used to define human MDSCs (33), we extended the characterization of these cells to include HLA-DR^−^ CD14^+^ monocytes in addition to the canonical CD33^+^ CD11b^+^ HLA-DR^−^CD14^+^ cell subset. Indeed, circulating monocytes expressing the monocytic CD14^+^ marker but lacking the expression of MHC class II cell surface receptor HLA-DR have also been identified as major mediators of tumor-induced immunosuppression (13, 34).

Our results demonstrate that the DNA-SIV + ALVAC-SIV + gp120 alum regimen increases the levels of M-MDSC-like cells (HLA-DR^−^ CD14^+^ cells) that are associated with an increased risk of SIV_mac251_ acquisition. The frequency of MDSCs and their transcriptome were associated with a reduction of interferon-stimulated genes (ISGs) and T and B cell pathways. Moreover, we found that an increase in arginase activity was inversely associated with protective classical monocytes and *NLRP3*. Arginase activity was instead positively associated with harmful CD16^+^ monocytes and, in turn, with a decrease in gag-specific IFN-γ^+^ and TNF-α^+^CD8^+^ T cell responses, and increased risk of SIV_mac251_ acquisition. These results unravel complex mechanisms of vaccine-induced protective immunity through the crosstalk between activating and suppressive myeloid cells.

## Materials and Methods

### Animal Study and Challenge

The study was conducted as previously described (32). All animals used in this study were colony-bred rhesus macaques (*Macaca mulatta*) provided by Covance Research Products. Monkeys were housed and handled in accordance with the standards of the Association for Assessment and Accreditation of Laboratory Animal Care International, and the care and use of the animals complied with all relevant institutional (U.S. National Institutes of Health) guidelines. The protocol (AUP 491) was approved by the Advanced BioScience Laboratories Institutional Animal Care and Use Committee.

**Figure 1:**
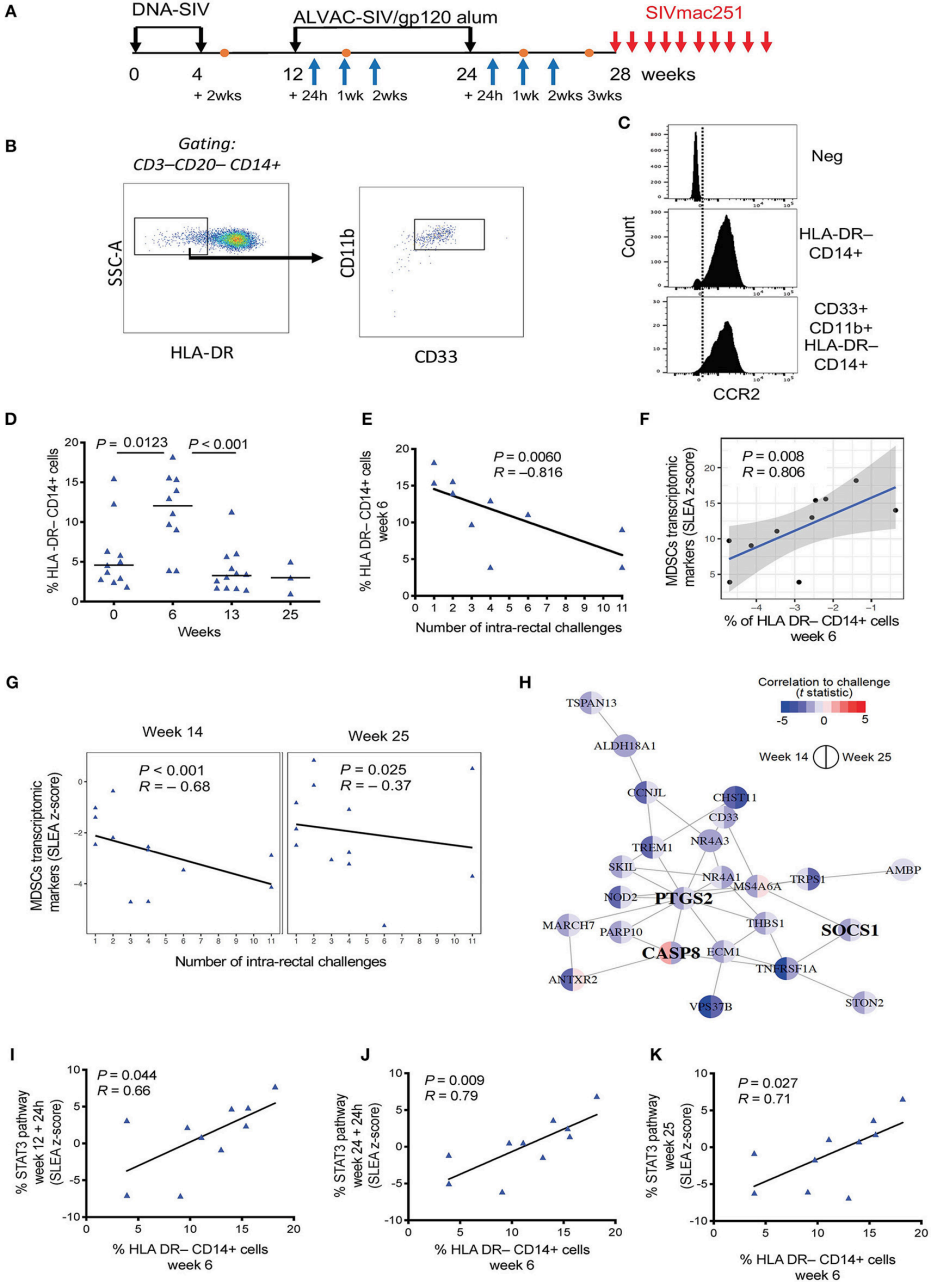
**(A)** Vaccine strategy. Orange dots indicate the collection of PBMCs, and blue arrows indicate collection of whole blood for microarray analysis. **(B)** Phenotypic identification of M-MDSC (CD33^+^ CD11b^+^ HLA-DR^−^ CD14^+^) and MDSC-like cell (HLA-DR^−^ CD14^+^) subsets in the blood of macaques. Data obtained from a naïve, non-vaccinated animal is shown. **(C)** Histogram showing CCR2 positivity in the HLA-DR^−^ CD14^+^ population and CD33^+^CD11b^+^ HLA-DR^−^ CD14^+^ cells. The isotype control is shown in the first panel. **(D)** Percentage of HLA-DR^−^ CD14^+^ cells in the blood of vaccinated macaques collected before vaccination and after each immunization (week 0, *n* = 11; week 6, *n* = 10; week 13, *n* = 12; week 25, *n* = 4). Mean and standard error are shown for each group. **(E)** Correlation between the level of HLA-DR^−^ CD14^+^ cells at week 6 in 10 vaccinated animals and the number of challenges to infection. **(F)** Scatterplot showing the levels of MDSC transcriptomic markers at week 14 as a function of HLA-DR^−^ CD14^+^ measured at week 6. Sample enrichment analysis was used to average the expression of MDSC transcriptomic markers. Spearman correlation and *t*-test was used to find the correlation between MDSC transcriptomic markers and HLA-DR^−^ CD14^+^ cells. **(G)** Scatterplot representing the average expression of MDSC transcriptomic markers at 2 weeks after the 1st ALVAC boost (week 14) and 1 week after the second ALVAC boost (week 25) as a function of the number of SIV challenges to infection for all 12 vaccinated animals. Linear regression (black line) and the Spearman correlation are shown on each plot. **(H)** MDSC transcriptomics markers associated with increased risk of SIV acquisition following challenge (i.e., leading edges of the GSEA analysis) were used as input to infer gene-to-gene network using the GeneMANIA application. Each node corresponds to an MDSC marker correlated with acquisition at week 14 or/and week 25. The color of the node is proportional to the t statistic testing that the correlation between the gene and challenge is different from zero. Edges are inferred by GeneMANIA based on co-expression, co-localization, genetic interaction or physical protein-protein interactions. MDSC markers that are interferon-stimulated genes (ISG) are labeled in bold. **(I–K)** Sample level enrichment analysis of *STAT3* pathways showing positive correlation with the levels of HLA-DR^−^ CD14^+^ cells at week 6.

Twelve juvenile macaques were immunized intramuscularly twice with DNA-SIV at weeks 0 and 4 (Figure 1A) as previously described (35). Each immunization contained a total of 6 mg of DNA in 1.5 ml PBS. DNA primed animals were given the following DNA constructs: 206S SIV p57gagmac239 (1 mg); 209S MCP3-p39gagmac239 (1 mg); 221S SIV_macM766_ gp160 (2 mg); 103S LAMP-Polmac239 (2 mg). At weeks 12 and 24, all macaques were boosted with intramuscular inoculations of 10^8^ p.f.u. of ALVAC recombinants (vCP2432), expressing SIV_mac251_ gag-pro and gp120TM (Sanofi Pasteur), and with 200 μg each of SIV_mac251−M766_ and SIV_smE660−CG7V_ gp120-gD proteins adjuvanted in alum alhydrogel (InvivoGen), as previously described (36). The proteins were administered intramuscularly in the thigh opposite the one of the ALVAC injection site. In addition to the 12 vaccinated animals, 6 concurrent control animals were treated with the alum adjuvant at weeks 12 and 24. Four weeks after the last immunization (week 28), the 12 immunized macaques and 6 control animals were challenged intrarectally with 10 repeated low-doses of pathogenic SIV_mac251_ (120 TCID50, 50% tissue culture infective dose) once a week. Thirty-five non-contemporaneous controls, challenged with the same virus stock in the same facility and following the same procedures, were added to the 6 concurrent controls as previously described (32). The time of acquisition was identified as the number of exposures to SIV_mac251_ prior to the detection of SIV-RNA in plasma.

### Measurement of SIV Viral DNA in Rectal Tissue

SIV_mac251_ DNA was quantified in mucosal tissues collected 2–3 weeks after viral infection. Genomic DNAs were isolated from tissues and the absolute quantitation of pro-viral DNA load was assessed by a real-time qPCR assay with sensitivity up to 10 copies × 10^6^ cells, as previously described (37).

### FACS Staining

Identification of M-MDSCs was performed together with monocyte subsets. PBMCs (5–10 × 10^6^ cells) were stained with the following antibodies: CD3 (clone SP34-2; BD Biosciences; Catalog #563916, 1.0 μl) and CD20 (clone 2H7; BD Biosciences; Catalog #560735, 1.0 μl), both in PE-Cy7, and NHP-CD45-BV786 (clone D058-1283; BD Biosciences; Catalog #563861, 3.0 μl), CD14-APC (clone M5E2; BD Biosciences; Catalog# 561390, 7.5 μl), CD16-FITC (clone 3G8; BD Biosciences; Catalog #555406, 5.0 μl), HLA-DR-APC-Cy7 (clone L243; BioLegend; Catalog #307618, 4.0 μl), CD11b-Pe-Cy5 (clone ICRF44; BioLegend; Catalog #301308, 0.0625μl), CD33-PE (clone AC104.33; Miltenyi Biotec; Catalog #130-091-732, 5.0 μl), CD192 (CCR2)-BV421 (clone 48607; BD Biosciences; Catalog #564067, 3.0 μl), and CD184 (CXCR4)-PE-CF594 (clone 12G5; BD Biosciences; Catalog #562389, 5.0 μl). Aqua LIVE/DEAD kit (Invitrogen; Catalog #L34966, 3.0 μl) was used to exclude dead cells. For this identification panel, myeloid cells were gated as CD45^+^Lin^−^ (CD3 & CD20). Monocyte populations were identified and classified by the expression of CD14 and CD16 as Classical monocytes: Lin^−^CD45^+^CD14^+^CD16^−^HLA-DR^+^; Intermediate: Lin^−^CD45^+^CD14^+^CD16^+^ HLA^−^DR^+^; and Non-Classical: Lin^−^CD45^+^CD14^−^CD16^+^HLA-DR^+^. Flow cytometry acquisition was performed on an LSRII (BD Biosciences) with a minimum of 500,000 events recorded. Marker expression was examined using FACSDiva software (BD Biosciences) and further analyzed using FlowJo v10.1 (Treestar, Inc., Ashland, OR).

### Kynurenine and Tryptophan Plasma Levels

Tryptophan and Kynurenine plasma concentrations were measured by using the Tryptophan ELISA (Rocky Mountain Diagnostics, Colorado Springs, CO, USA, Catalog #BA E-2700) and Kynurenine ELISA commercial kits (Rocky Mountain Diagnostics, Colorado Springs, CO, USA, Catalog #BA E-2200). For tryptophan measurement, 20 μl of plasma were precipitated, the recovered supernatants were derivatized, and the product was used to perform the ELISA according to manufacturer instructions. For kynurenine assay, 10 μl of plasma were acylated and used to perform the ELISA according to manufacturer instructions. The data are presented as the ratio between kynurenine and tryptophan (Krn/Try) levels.

### Intracellular Staining

PBMCs (1–3 × 10^6^ cells) were stimulated with 2 μg ml^−1^ of the cognate peptide pools for 6 h in RPMI containing 10% human serum in the presence of 5 μg ml^−1^ of GolgiPlug (10 μg ml^−1^, BD Biosciences). Negative controls received an equal concentration of DMSO. Cells were stained with the following surface marker-specific antibodies for 30 min at 4°C: APC Cy7 anti-CD3 (SP34.2; BD Biosciences), BV421 anti-CD4 (OKT4; BioLegend), and CD8-BV570 (clone RPA-T8; BioLegend). Following fixation and permeabilization, cells were stained with ECD anti-CD69 (clone TP1.55.3; Beckman Coulter), PE anti-IL-2 (MQ1-17H12; BD Biosciences), IFNγ-APC (B27; BD Biosciences), and FITC anti-TNFα (Mab11; BD Biosciences). The Aqua LIVE/DEAD kit (Invitrogen) was used to exclude dead cells. Samples were acquired on an LSRII flow cytometer and analyzed using FlowJo version 9.6.3 (Treestar, Inc.).

### Luminex

Cryopreserved supernatants were analyzed using three MILLIPLEX Non-Human Primate Multiplex assays (EMD Millipore). The following targets were assayed following the manufacturer's instructions: IL-1β, IL-2, IL-4, IL-6, IL-8, IL-10, IL-13, IL-17, IFN-γ, MCP-1, MIP-1a (PRCYTOMAG-40K-11), IL-21, IL-22, IL-23, RANTES (PRCYT2MAG-40K-04), and TGF-β (TGFBMAG-64K). After thawing the samples on ice, 25 μl of each supernatant was briefly loaded into the well and mixed with 25 μl assay buffer and 25 μl magnetic beads. The plates were incubated under agitation at 4°C for 18 h. After washing, 25 μl of detection antibody were added to each well and incubated for 1 h at room temperature (RT). Next, 25 μl streptavidin-PE was added to each well and incubated for 30 min at RT. Finally, wells were washed and 150 μl sheath fluid was added. Samples were acquired on a Bio-Plex 200 System (Bio-Rad).

### Arginase Activity

Arginase activity was analyzed on Plasma using the Arginase Activity Assay Kit (MAK112, Sigma-Aldrich, St. Louis, MO) following the manufacturer instructions. Briefly, samples were thawed on ice and, in order to deplete the urea, 50 μl of plasma were loaded in an Amicon®Ultra 10K centrifugal filter (UFC501096 EMD Millipore), diluted with pure water to 500 μl, and centrifuged at 13,000 × g for 30 min at 4°C. Following centrifugation, the eluted solution was discarded. Filtered samples were then diluted with pure water to 500 μl, and centrifuged at 13,000 × g for 30 min at 4°C. At the end of centrifugation, the remaining volume of each sample was measured, and ultra-pure water was added to reach a final volume of 40 μl. Each sample was loaded into 2 wells of a 96-well plate (20 μl/well), representing the sample well and the sample blank well, and 20 μl/well of ultra-pure water were added to each well. Together with samples, the plate was loaded with urea standard and water as positive and negative controls, respectively. Samples were loaded in singlicate , whereas controls were loaded in duplicate. Ten microliter of 5X substrate buffer, composed of Arginine Buffer and Mn Solution, were added to the wells except for sample blank wells, and they were incubated for 120 min at 37°C. Following the incubation, 200 μl of Urea Reagent, composed of Reagents A and B, was added to each well to stop the reaction. Finally, 10 μl of 5X Substrate Buffer was added to the sample blank wells to have the same reagents proportion of reagents as the Sample Wells. After mixing, the plate was incubated for 60 min at RT, and finally acquired with microplate spectrophotometer Power Wave XS2 (BioTek Instruments, Winooski, VT) to measure the absorbance at 430 nm (A_430_) of each well. The arginase activity was determined per the following equation.

$$\begin{array}{ll} Arginase\;Activity & =\\ \left[\frac{\left(A_{430}\;sample\;well\right)-\left(A_{430}\;sample\;blank\;well\right)}{\left(A_{430}\;urea\;standard\right)-\left(A_{430}\;water\right)}\right]\\ \times & \left[\frac{1\text{mM }\times \;50\times 10^{3}}{40\mu l\times 120\text{ min}}\right] \end{array}$$

### Reactive Oxygen Species (ROS) and Reactive Nitrogen Species (RNS) Analysis

The total free radical contents were analyzed on Plasma and mucosal cell supernatants using the OxiSelect™ *in vitro* ROS/RNS Assay Kit (Cell Biolabs, Inc., San Diego, CA, USA, Catalog #STA-347) following manufacturer instructions. Briefly, cryopreserved samples were thawed in ice, and insoluble particles were removed by centrifuging at 10,000 g for 5 min. Following this, 50 μl of standards, plasma diluted 1:5 with PBS, or undiluted mucosal cell supernatants were single-loaded in a 96-well plate suitable for fluorescent measurement. To each well was then added 50 μl of Catalyst, incubated for 5 min at room temperature, and then 100 μl of dichlorodihydrofluorescein (DCFH) solution. The plates were incubated at RT for 30 min in the dark and the fluorescence was read using a plate reader at 480 nm excitation/530 nm emission (VictorX4, Perkin Elmer, Inc., Waltham, MA, USA). The ROS/RNS content of each sample was determined by interpolation of unknown samples with a standard curve generated with hydrogen peroxide. For plasma samples, the standard curve was generated by diluting the standards with PBS. For mucosal cell supernatants, the standard curve was generated by diluting the standards with R10 media.

### Gene Expression Array Analysis

Twelve macaques vaccinated with DNA prime and ALVAC/gp120 alum boost were included in a gene expression profiling study. PreAnalytiX tubes (#762165) were used to collect 2.5 ml of whole blood from these animals at 24 h and 2 weeks after the 1st boost or 1 week after the second boost. Paxgenes were gently rocked for 2 h and then stored at −80°C. Total RNA was extracted using Agencourt RNAdvance Blood Kit (Beckamn Coulter #A35604). The isolated total RNA was checked for quantity and quality using a NanoDrop 2000c (Thermo Fisher Scientific) and an automated electrophoresis system (Experion, Bio-Rad). Samples with an RQI classification ≥7.0 were selected to proceed downstream to amplification. Samples were normalized at 50 ng for input and amplified using Illumina TotalPrep RNA amplification kits (Ambion) according to the manufacturer's protocol. Microarray analysis was conducted using biotinylated cRNA hybridized to Human HT-12 version 4 BeadChips (Illumina). The arrays were scanned using iSCAN (Illumina) and quantified using Genome Studio (Illumina). Analysis of the Genome Studio output data was conducted using R/Bioconductor software packages. Bead arrays were read, and missing values (>0.01%) were imputed using the nearest-neighbor method as implemented in the R package impute. Quantile normalization and log2 transformation for variance stabilization were then applied to raw intensities.

For each gene, a linear regression model with the number of SIV challenges to infection as an independent variable and gene expression as a dependent variable was fit using the R package LIMMA. A moderated *t*-test was used to test that the coefficient of regression was statistically different from 0. The Benjamini–Hochberg method was used to correct the *P-*values for multiple testing (adjusted *P-*values). Genes with an adjusted *P-*value below 5% were defined as differentially expressed genes. GSEA was used to evaluate the gene sets (pathways) associated with the number of SIV challenges to infection and frequency of HLA-DR^−^ CD14^+^ measured at week 6. In GSEA, the most variable probes across samples were used to remove redundant probes annotated to the same gene. Genes were pre-ranked by LIMMA t statistic, and GSEA was used to assess the enrichment of gene sets from the Molecular Signatures Database gene sets (version 5.1) and transcriptomic markers of MDSCs (38). The GSEA Java desktop program was downloaded from the Broad Institute (http://www.broadinstitute.org/gsea/index.jsp) and used with GSEA Pre-Ranked module parameters (number of permutations: 1,000; enrichment statistic: weighted; seed for permutation: 111; 15 ≤ gene set size ≤2,000). Sample-level enrichment analysis was used to investigate the enrichment of pathways in the different samples. Briefly, the expression of all the genes in a specific pathway was averaged across samples and compared to the average expression of 1,000 randomly generated gene sets of the same size. The resulting Z score was then used to reflect the overall perturbation of a pathway in a sample.

### Network Analysis

GeneMANIA version 3.5.1 was used to identify relations (co-expression, co-localization, genetic interactions and physical interactions) between MDSC transcriptomic marker. To that end, the human orthologs and homologs of the macaque's genes included in the classifier were obtained from the NCBI gene and homologene portal. The human homologs were then imported into GeneMANIA, and a network was generated with default parameters (equal weight of network) except no (0) inferred nodes were used to consolidate the network.

### Statistical Analysis

The Mann-Whitney-Wilcoxon test was used to compare continuous factors between the two groups. Correlation analysis was performed using the Spearman rank correlation method using exact permutation *P-*values. Multiple comparison analysis were performed to include all the time points analyzed using the Benjamini-Hochberg or the Tukey's multiple comparison analysis when no association between the frequency of these cells was found at different timepoints.

## Results

### The DNA-SIV Prime Induces HLA-DR^−^ CD14^+^ Cells That Correlate With an Increased Risk of SIV_mac251_ Acquisition

CD14^+^ cells with low or absent HLA-DR expression have been linked to suppressive monocytic function (34), and they have recently been characterized as myeloid-derived suppressor cells in rhesus macaques (31). The DNA-prime ALVAC + gp120 alum boost strategy demonstrated a significant 52% vaccine efficacy in protecting macaques against SIV_mac251_ (32). Here we assessed the kinetics of monocytic-MDSCs and their role in this protection. Blood was collected pre-vaccination, 2 weeks after the prime (2xDNA, week 6), and after each immunization with ALVAC + gp120 alum (boosts at weeks 13 and 25; Figure 1A). Circulating monocytic MDSCs were identified as live HLA-DR^−^ CD14^+^ cells that were negative for CD3 and CD20 molecules (lineage). Although conflicting reports have arisen on the validity of including CD33 as a marker for macaques MDSC (31), we also took into consideration the CD33^+^ CD11b^+^ HLA-DR^−^ CD14^+^ cell population (referred to as M-MDSCs). The gating strategy used to identify M-MDSCs and HLA-DR^−^ CD14^+^ cells in the blood of a non-vaccinated animal is shown in Figure 1B. Both identified subsets were highly positive for the CCR2 marker, in line with phenotypic markers used to define MDSCs in humans (Figure 1C).

We could not detect significant changes in the levels of circulating CD33^+^ CD11b^+^ HLA-DR^−^ CD14^+^ cells during the course of immunization, possibly due to the high variability observed in this subset in addition to the relatively small number of animals in this group (Supplementary Figure 1A). Interestingly, the frequency of HLA-DR^−^ CD14^+^ cells in blood was significantly increased by the DNA-prime (baseline vs. week 6: *P* = 0.0123, one-way ANOVA, Tukey's multiple comparisons), while no differences were detected between the frequencies pre-vaccination and after the 2nd ALVAC + gp120 boosts (Figure 1D). Of note, there was no association between the frequency of these cells at different timepoints. Strikingly, we observed a significant association between the frequency of HLA-DR^−^ CD14^+^ cells after the DNA prime (week 6) and the number of challenges to infection (*P* = 0.0006, *R* = −0.816, Spearman test (Figure 1E). Significance was retained when the *P* value was adjusted for the 4 time points analyzed (Benjamini-Hochberg test, *P* = 0.0160).

Total blood was collected for microarray analysis before and at 24 h, 1 or 2 weeks after the first boost (week 12 + 24 h and week 13 and 14), and 24 h and 1 and 2 weeks after the second boost (week 24 + 24 h, and weeks 24 and 25) with the ALVAC-SIV + gp120-alum (Figure 1A). Transcriptomic signatures of vaccine-induced immune responses were identified as changes in gene expression after the vaccination compared to the pre-vaccination timepoint. To determine whether our vaccine induced MDSCs associated genes, Gene set enrichment analysis (GSEA) was used and vaccine induced genes were compared to a MDSC-associated genset previously identified by Heim et al. (38) (Supplementary Figure 1B). Transcriptomic markers of MDSCs were significantly induced at 24 h after each boost with ALVAC + gp120, as shown in Supplementary Table 1. Vaccine-induced MDSC markers included *PTGS2*, the gene coding for the enzyme cyclooxygenase 2 (*COX2*). Of note, the vaccine-induced transcriptomic markers of MDSCs measured at 2 weeks following the 1st boost (week 14) were positively associated with the frequency of HLA-DR^−^ CD14^+^ cells (*P* = 0.008, *R* = 0.806; adjusted *P* = 0.19; Figure 1F). Association between transcriptomic markers of MDSCs induced by vaccination and the number of SIV challenges was then assessed (Supplementary Table 2). In addition to the enrichment of MDSC transcriptomic markers among genes associated with SIV_mac251_ acquisition, the average expression of MDSC transcriptomic markers measured after each boost was significantly negatively correlated with the number of challenges (week 14: *P* < 0.001, *R* = −0.68; week 25: *P* = 0.025, *R* = −0.37 by the Spearman test, and *P* = 0.049 when the Benjamini-Hochberg correction is applied; Figure 1G). *CD33* and of cyclooxygenase-2 (*COX-2 or PTGS2)* were among the MDSC genes associated with an increased risk of SIV_mac251_ acquisition after the 1st boost (week 14), as shown in the network analysis (Figure 1H) and in Supplementary Table 2. Together with PGE_2_, the expression of *COX-2* may represent a critical step for redirecting dendritic cell development toward functionally stable MDSCs (39). Indeed, PGE_2_ together with MDSC-inducing factors IL-1β and IFNγ induce high levels of *COX-2* in differentiating MDSCs and stabilizing their suppressive functions (39).

All together, these results suggest that the DNA-SIV-induced HLA-DR^−^ CD14^+^ cell population may be enriched with M-MDSCs. Thus, we will refer to this population as M-MDSC-like cells.

In line with this observation, the frequency of M-MDSC-like cells was also positively correlated with activation of the *STAT3* signaling pathway at 24 h after the first (week 12 + 24 h: *P* = 0.044, *R* = 0.66) and second boosts (week 24 + 24 h: *P* = 0.009, *R* = 0.79), and at week 25 (*P* = 0.027, *R* = 0.71; Figures 1I–K).

### Plasma Arginase Level Is Associated With an Increased Risk of SIV_mac251_ Acquisition

**Figure 2:**
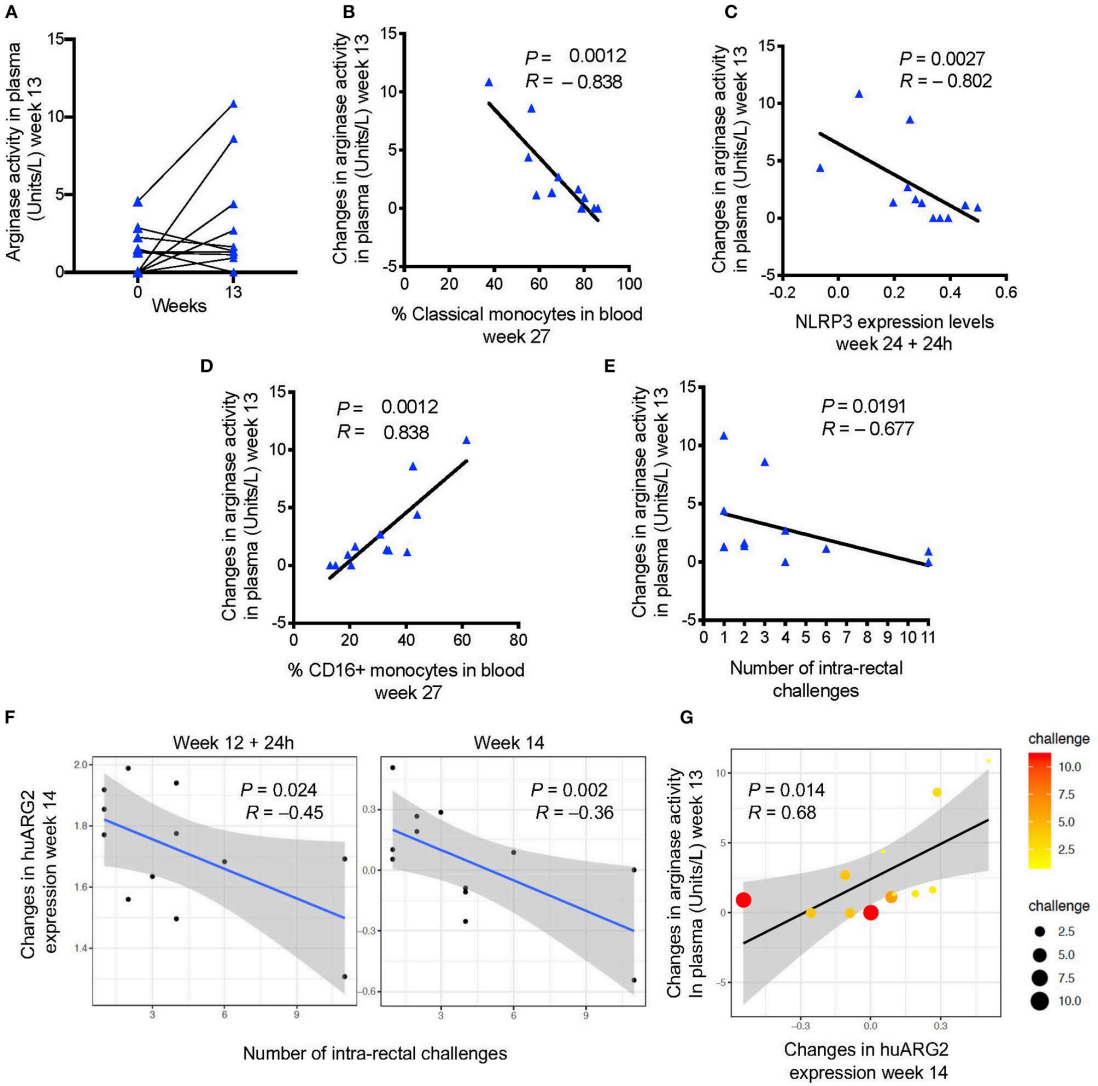
**(A)** Arginase activity measured before and after immunization (baseline or week 0 and week 13). **(B)** Changes in arginase activity (week 13—pre-vaccination) in plasma are negatively associated with **(C)** the frequency of classical monocytes in blood (week 27), and with *NLRP3* expression (24 h after the last immunization), and **(D)** positively associated with the frequency of CD16^+^ monocytes. **(E)** Vaccine induced levels of arginase activity and **(F)** probe annotated to human *ARG2* are negatively associated with the number of challenges to infection (increased risk of SIV acquisition). **(G)** A positive correlation was found between vaccine induced arginase activity and *ARG2* gene expression. Scatter plot shows both gene and plasma levels in relation to the number of challenges (dot size and color). In all the plots, 12 animals are shown.

Arginine metabolism plays a central role in the regulation of immune cell function (40). MDSCs expressing arginase and an increase in arginase activity have been described in trauma, cancer, and in certain infections (41). We measured arginase activity in the plasma of non-vaccinated macaques and macaques vaccinated after the first ALVAC + gp120-alum boost (week 13). Arginase activity levels were increased in some animals, though the overall increase was not significant (Figure 2A). However, changes in the arginase activity levels after the first boost (week 13 levels—pre-vaccination levels) were negatively associated with the frequency of classical monocytes, and with the *NLRP3* inflammasome pathway, two previously identified correlates of HIV vaccine protection (32) (arginase vs. classical monocytes: *P* = 0.0012, *R* = −0.838; arginase vs. *NLRP3* expression: *P* = 0.0027; *R* = −0.802; Figures 2B,C). On the contrary, vaccine-induced arginase activity was positively associated with the frequency of CD16^+^ monocytes, a previously identified correlate of increased risk of SIV_mac251_ acquisition (32) (arginase vs. CD16^+^ monocytes: *P* = 0.0012, *R* = 0.838; Figure 2D). Accordingly, the arginase activity levels were also associated with increased risk of SIV_mac251_ acquisition (Spearman test: *P* = 0.019; *R* = −0.67; Figure 2E).

In MDSC, L-arginine is metabolized by two enzymes: a cytoplasmic arginase I (ARG1), and a mitochondrial arginase II (ARG2) that is widely expressed and associated with control of NO production (41). While no probe matched the arginase genes for rhesus macaques on the microarray platform, probes annotated to human *ARG2* (*huARG2*) there was negative association with acquisition (Figure 2F) in vaccinated animals at week 14 (*P* = 0.078, *R* = 0.658, Benjamini-Hochberg correction), and with arginase levels at week 13 (*P* = 0.014; *R* = 0.68; Figure 2G), but did not withstand correction for multiple comparisons on the 4 point analyzed was included (*P* = 0.693).

### Nitric Oxide-Related Genes Correlate With Plasma Arginase Level and ARG2 Expression

**Figure 3:**
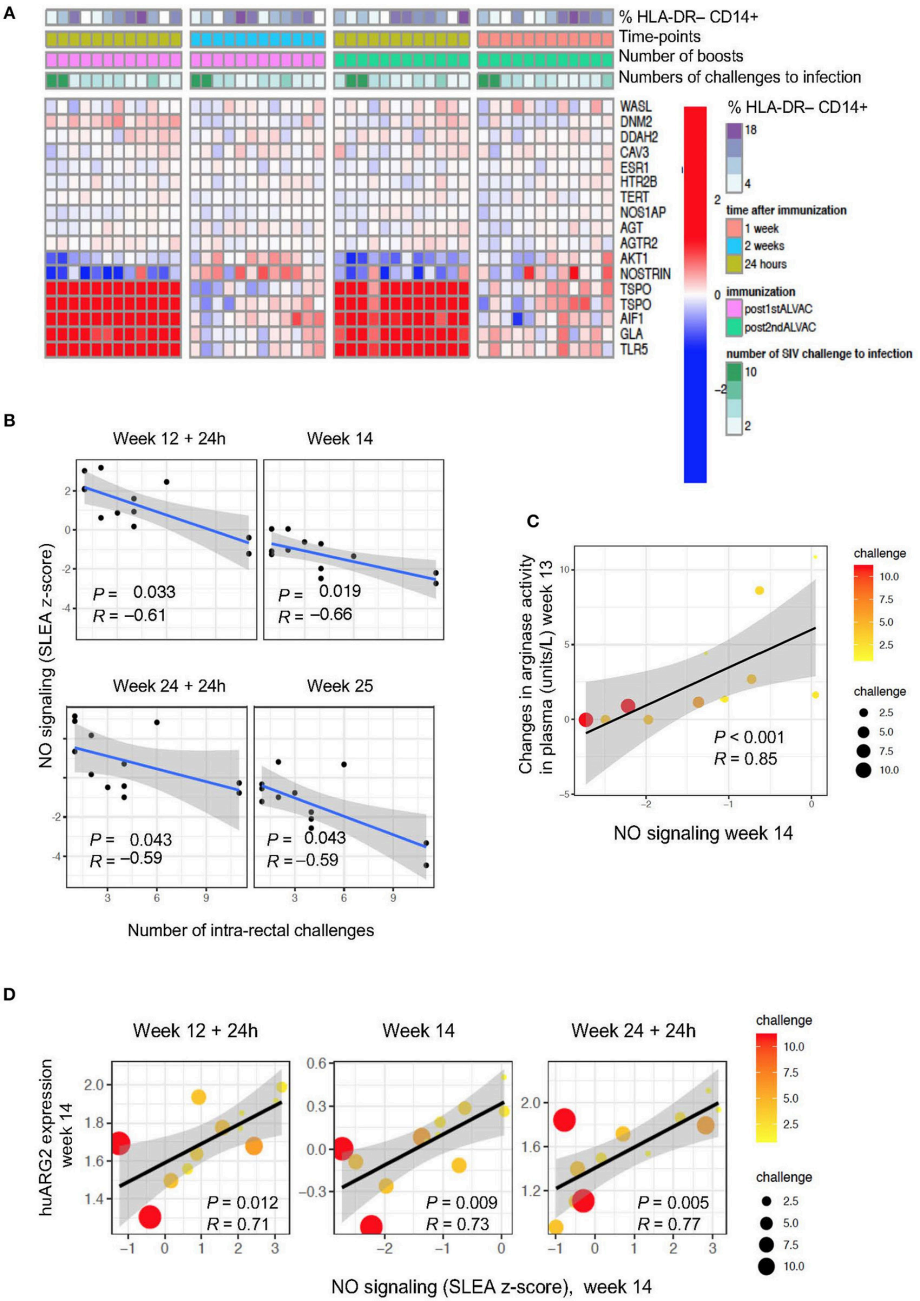
**(A)** Heat map showing gene changes in NO pathways significantly associated with virus acquisition. GSEA revealed an enrichment of NO-related genes correlated with challenge. The log2-fold difference (post-vaccination—pre-vaccination) of the NO-related genes (leading edge genes of the gene sets GO_REGULATION_OF_NITRIC_OXIDE_SYNTHASE_ACTIVITY and GO_REGULATION_OF_NITRIC_OXIDE_BIOSYNTHETIC_PROCESS) is presented in the heatmap. **(B)** NO pathway association with number of challenges to infection. Sample enrichment analysis was used to average the expression of the NO-related genes for each sample collected post-vaccination (y-axis) and is presented as a function of the number of SIV challenge to acquisition (x-axis). A linear regression model was fitted (blue line), and its 95% confidence interval is presented (gray zone). A Spearman correlation and *t*-test were used to assess the significance of the association between NO gene expression and challenge. **(C)** Scatterplot of arginase activity as a function of NO gene expression at week 14. The size of the dots is proportional to the number of SIV challenges to acquisition for each animal. **(D)** Scatterplot of the gene expression of hu*ARG2* as a function of NO gene expression at different timepoints post-boosts. The size of each point is proportional to the number of SIV challenges to acquisition for each animal.

The regulation of arginine availability is a mechanism that can potentially lead to the control of NO production (42). Indeed, through arginine depletion, MDSCs may control NO production and regulate other arginine-dependent biological processes. We attempted to measure NO and intracellular iNOS expression levels by ELISA and FACS analysis, respectively, but we were unable to find antibodies that cross-react with rhesus macaques. Hence, we performed pathway enrichment analysis on total blood collected at 24 h and 1 week or 24 h and 2 weeks after the two ALVAC boosts (weeks 12, 14, 24, and 25; Figure 3A). Genes implicated in synthesis and signaling pathways of nitric oxide were associated with SIV_mac251_ acquisition (Supplementary Table 3; GSEA: nominal *P-*value ≤0.05) at 4 time points: at 24 h and 1 or 2 weeks after the 3rd and 4th immunization (Figure 3B), however there was no significance when all 4 time points were considered. NO-related genes associated with SIV acquisition include *NOS1AP* (the adaptor protein of the NO synthase), *NOSTRIN* and *AGTR2* (coding for the inhibitors of endothelial NO synthase), and *AKT1* (coding for a kinase regulated by NO). Interestingly, NO-related genes induced by vaccination at week 14 (2 weeks after the 1st ALVAC + gp120 alum boost) were positively associated with the changes in arginase levels in plasma following the same immunization (week 13) (*P* < 0.001, *R* = 0.85). NO biosynthesis may be at least partially regulated by *Arg2* (43), and the NO pathway was also significantly associated with *huARG2* expression (Figure 3D). All together, these results suggest that vaccination-induced changes in NO related genes and plasma arginase levels affected protection against SIV acquisition.

### HLA-DR^−^ CD14^+^ Cells Are Associated With Decreased Expression of Interferon-Stimulated Genes and T Cell Pathways

**Figure 4:**
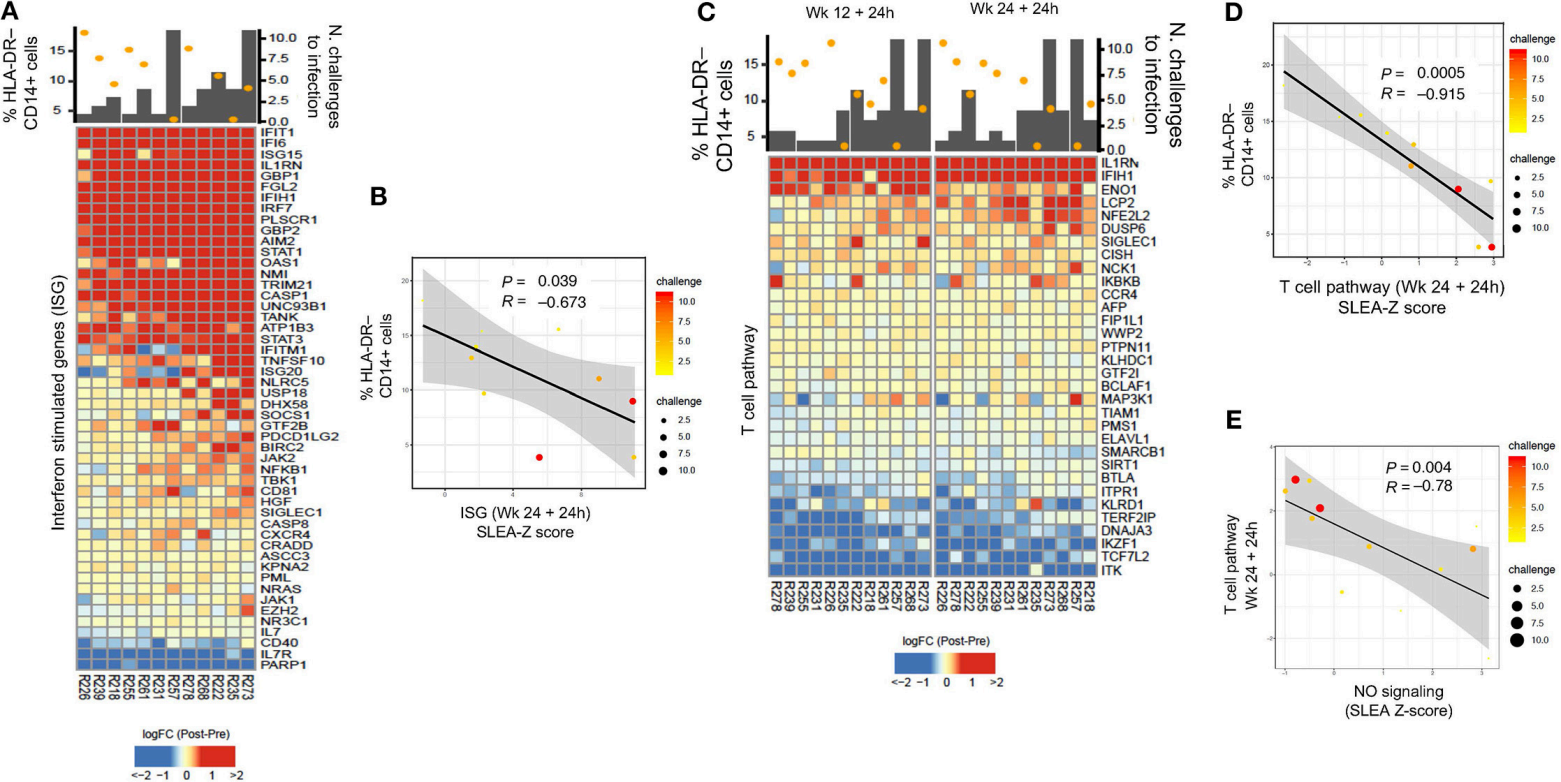
**(A)** Heatmap showing log2-fold change (post-vaccination—pre-vaccination) of interferon-stimulated genes 24 h after the 2nd ALVAC boost negatively correlated with HLA-DR^−^ CD14^+^ cell frequencies measured 2 weeks after prime, and positively correlated with protection from SIV challenge. **(B)** Sample enrichment analysis was used to summarized the expression of interferon-stimulated genes per sample. The scatter plot shows the frequencies of HLA-DR^−^CD14^+^ cells as a function of the interferon-stimulated genes measured 24 h after the 2nd boost. **(C)** Heatmap showing log2-fold change (post-vaccination—pre-vaccination) T cell pathway genes that are negatively correlated with HLA-DR^−^ CD14^+^ cells and, at the same time, with protection from SIV acquisition. **(D)** The scatter plot shows the frequencies of HLA-DR^−^CD14^+^ cells as a function of the T cell pathway measured 24 h after the 2nd boost **(E)** and the T cell pathway as a function of the NO pathway. In all the scatterplots, the size of the dots is proportional to the number of SIV challenge to infection of the vaccinated animals. A Spearman correlation and *t*-test was used to test the correlation is different from 0.

Because MDSCs have been implicated in the suppression of interferon stimulating genes (ISGs) and adaptive immune responses, we first looked for possible associations between their levels and specific T cell responses (Supplementary Table 3). Transcriptomic analysis revealed a negative correlation between the level of M-MDSC-like cells measured at prime, and ISGs that underwent a log2-fold change (Figure 4A). Following the second boost with ALVAC + gp120 alum, the expression of ISGs correlated with a decreased risk of SIV_mac251_ acquisition (*P* = 0.0136, *R* = 0.69, Figure 4A), and was negatively associated with the frequency of HLA-DR^−^ CD14^+^ cells at the prime (*P* = 0.0394, *R* = −0.673, sample-level enrichment analysis (SLEA) method, Figure 4B). Genes included the kinases *JAK1, JAK2*, the transcription factor *STAT1*, and the suppressors of cytokine signaling SOCS1, as shown by the network analysis in Figure 1H, which inhibit receptor signaling by directly inhibiting both JAK kinases and cytokine receptors (44, 45).

T cell pathways induced by vaccination were also found to be associated to protection (defined as increased number of challenges to infection) (24 h after the 1st boost *P* = 0.0007, *R* = 0.836, and 2nd boost *R* = 0.683, *P* = 0.0143, Figure 4C). At the same time, T cell pathways measured at 24 h after the 2nd boost were associated with the frequency of HLA-DR^−^ CD14^+^ cells at the prime (*P* = 0.0005, *R* = −0.915, Figure 4D). Of note, the NO pathway had a significant negative correlation with the same T cell activation pathways at 24 h after the 1st and 2nd boosts (Figure 4E, *P* = 0.00412, *R* = −0.78).

### HLA-DR^−^ CD14^+^ Cells Are Associated With Decreased Expression of B Cell Pathways

**Figure 5:**
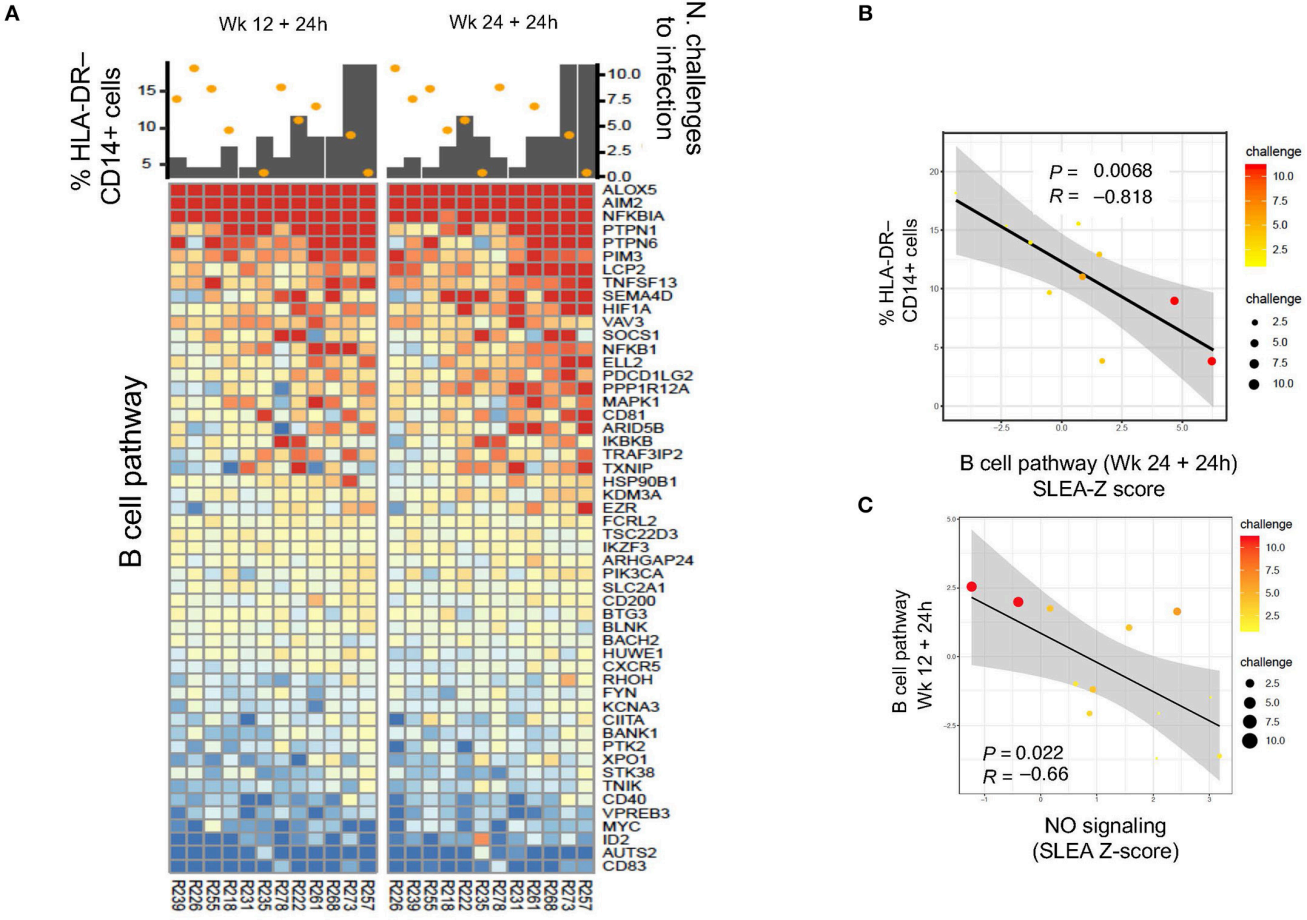
**(A)** Heatmap showing log2-fold change (post-vaccination—pre-vaccination) in B cell pathways that are negatively correlated with HLA-DR^−^ CD14^+^ cell frequency and also correlated with protection from SIV acquisition. **(B,C)** Sample enrichment analysis was used to summarized per sample the expression of genes part of the B cell pathway. The scatter plots shows the frequencies of HLA-DR^−^CD14^+^ cells as a function of the B cell pathway measured 24 h after the 2nd boost **(B)** and the B cell pathway as a function of the NO pathway **(C)**. The size of the dots is proportional to the number of SIV challenge to infection of the vaccinated animals. A Spearman correlation and *t*-test was used to test the correlation is different from 0.

We then asked the question whether an association could be found with B cell pathways (Supplementary Table 3). Indeed, B cell pathways were negatively correlated with M-MDSC-like cell frequencies at 24 h after the first boost (*P* = 0.035, *R* = −0.68, data not shown) and second boost (*P* = 0.0068, *R* = −0.818), and positively associated with protection (1st boost: *P* = 0.003, *R* = 0.78; 2nd boost: *P* = 0.065, *R* = 0.55; Figures 5A,B). Thus, these results suggest a harmful long-term effect of the prime on monocytic myeloid suppressive cells that decreases vaccine-induced protection. The NO pathway had also a significant negative correlation with the B cell activation pathway at 24 h after the 1st and 2nd boosts (Figure 5C: *P* = 0.022, *R* = −0.66).

### Arginase and ROS Levels Correlate With Reduced SIV-Specific CD8^+^ T Cell Responses

**Figure 6:**
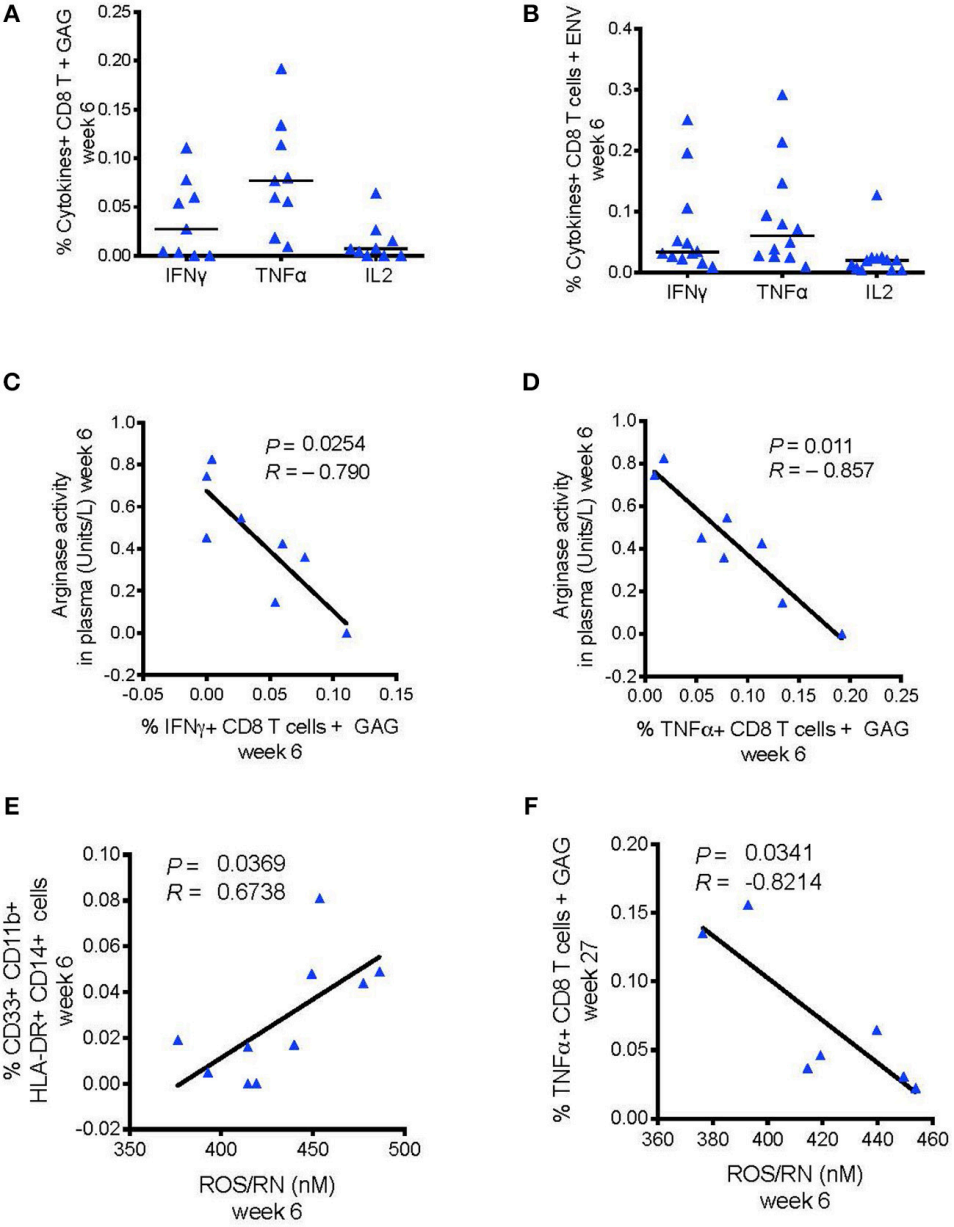
**(A)** CD8^+^ T cell responses measured in blood 2 weeks after the DNA-prime (week 6) by ICS. Cells were stimulated *in vitro* with overlapping peptides encoding for SIV_mac251_ env (*n* = 12) **(B)** or gag (*n* = 9). **(C)** Negative association between the levels of arginase activity in plasma at week 6 and the levels of CD8^+^ T cells producing, IFN-γ, and **(D)** TNF-α responses to SIV-gag peptides. **(E)** Association between the frequency of ROS/RN levels with CD33^+^CD11b^+^ HLA-DR^−^ CD14^+^ cells (*n* = 10), and **(F)** with SIV-gag specific TNF-α^+^ CD8^+^ T cell (week 27).

Recent research showed that MDSCs could inhibit HIV-specific CD8^+^ T cell responses in macaques vaccinated with an MVA-based HIV vaccine strategy (24). Priming with DNA-SIV resulted in low, but detectable, Envelope- and Gag-specific CD8^+^ T cells producing IFN-γ, IL-2, and TNF-α measured in blood at week 6 by intracellular staining (Figures 6A,B). We did not find a direct correlation between the frequency of these cytokine-producing T cells and the levels of MDSCs or M-MDSC-like cells at any timepoint during vaccination (Supplementary Table 3). However, IFN-γ^+^ and TNF-α^+^ CD8^+^ T cell responses to gag associated negatively with plasma arginase activity at the same time (week 6; IFN-γ: *P* = 0.025, *R* = −0.79; TNF-α: *P* = 0.011, *R* = −0.87; Figures 6C,D). Moreover, the level of reactive oxygen species (ROS) and reactive nitrogen species (RNS) did not associated with the levels of HLA-DR^−^ CD14^+^ cell population (data not shown), however it associated with an increased frequency of the CD33^+^ CD11b^+^ HLA-DR^−^CD14^+^ cell subset (*P* = 0.037, *R* = 0.67; Figure 6E). In turn, the latter subset assisted with reduced levels of TNF-α^+^ CD8^+^ T cell responses to gag at the end of the immunization regimen (week 27; *P* = 0.034, *R* = −0.82; Figure 6F). MDSCs can activate T regulatory cells that dampen T cell responses via catabolism of the essential amino acid tryptophan (Tryp), and accumulation of the kynurenine (Kyn) metabolite. The Kyn/Tryp ratio measured in the plasma of macaques vaccinated with the DNA and ALVAC + gp120 alum regimens had no association with suppressive myeloid cells, nor with SIV-specific T cell responses or viral outcome (Supplementary Table 4). Hence, these results point to the catabolism of L-arginine as an important mechanism of immunosuppression involved in the low level of protection afforded by this vaccine strategy, as both arginase and NO target this essential amino acid.

## Discussion

In recent years, new myeloid-derived suppressor cell subsets have been identified and characterized in inflammatory conditions and tumors (12). Accumulating evidence indicates an important role for MDSCs in controlling immune responses to pathogens (46). The expansion and activation of MDSCs during viral infection have been described as both detrimental and beneficial to the host. Through their immune suppressive function, MDSCs may, in fact, hamper host immune responses but conversely also limit inflammation and collateral tissue damage following an infection (46). In the case of HIV, MDSC mediated suppression of immune activation could reduce target cells for the virus (24, 47–49). Most of the studies aimed to underscore the relative contribution of MDSCs in HIV pathogenesis have described them as harmful, as MDSCs expand during untreated chronic infection and their levels are associated with disease progression (1, 50–53).While less is known about the role of MDSCs in vaccines, non-responsiveness to immunization has also been linked to MDSC expansion. Indeed, in a peptide-prime/modified vaccinia Ankara (MVA) boost vaccine regimen the M-MDSCs-like cells frequency was positively associated with set-point viral load, suggesting a negative role in protection from high viral replication (26).

Previously, we identified different monocytic myeloid subsets as correlates of increased and decreased risk of acquisition in the blood of macaques vaccinated with the DNA-SIV + ALVAC-SIV + gp120 alum regimen (32). Further, classical monocytes (HLA-DR^+^CD14^+^CD16^−^ cells) were associated with a decreased risk of SIV acquisition (32). The engagement of the myeloid compartment and the generation of a memory innate response following ALVAC immunization was most likely driven by the activation of the *NLRP3* inflammasome and the release of IL-1β. CD16^+^ monocytes and *STAT3* activation correlated with increased SIV_mac251_ acquisition (32).

We postulated that immunosuppression by MDSCs may be playing a role in the limited vaccine efficacy (VE = 52%) afforded by the DNA-SIV + ALVAC-SIV + gp120 alum vaccine. In fact, the recombinant ALVAC vaccine vector is a known inducer of GM-CSF and CCL2 (54), and the common receptor CCR2 is expressed on virtually all classical monocytes and MDSCs. Vaccination induced myelopoiesis, and high levels of CCL2 were also detected after the DNA prime (32). Additionally, the DNA prime, the recombinant ALVAC vector, and the alum adjuvant are all known inflammasome activators, which in turn contributes to MDSC activation (9, 55, 56).

We observed that the HLA-DR^−^CD14^+^ cell population expanded after the DNA-prime. While the antibody panel we used was designed to detect M-MDSCs, the CD15 antibody clone we used showed limited cross-reactivity. Consequently, we cannot discount the possibility that some of the gated cells in the HLA-DR^−^CD14^+^ population are in fact neutrophils (31, 57). Unlike the study conducted by Lin et al., we did detect CD33^+^ cells within the HLA-DR^−^ CD14^+^ cell population in macaque PBMCs, in alignment with the findings of Sui et al. (24, 31). However, this population's frequency did not change during vaccination, nor did it associate with MDSC-related genes or *STAT3*. Altogether, our data strongly suggest that HLA-DR^−^ CD14^+^ cells may be enriched in M-MDSCs, as we found their frequency to associate positively with transcriptomic markers of MDSCs (38).

Vaccine-induced HLA-DR^−^ CD14^+^ cells, MDSC gene expression, and levels of *STAT3* pathway activation (32) were all correlates of increased risk of SIV acquisition, suggesting that MDSCs harm vaccine effectiveness. Of the four MDSC-mediated immunosuppressive mechanisms we studied, we identified the arginase catabolism and NO biosynthesis as the ones primarily associated with diminished protection of the DNA-SIV + ALVAC-SIV + gp120-alum vaccine. Vaccination with this regimen induced changes in the levels of arginase activity in the plasma, and animals with increased levels proved more susceptible to infection. In addition, a heightened level of *Arg2* expression was also associated with decreased vaccine efficacy. The physiological function of Arginase 2 in humans is still poorly understood, but studies have suggested a role in regulating cell arginine concentrations by controlling substrate availability for the biosynthesis of NO, proline, and polyamines from the arginine precursor (43). In fact, *Arg2* expression levels were associated with NO-related genes encoding for NO synthesis and signaling that associated with increased virus acquisition. We could not directly link arginase activity or NO pathway activation to HLA-DR^−^CD14^+^ cells, nor can we exclude the possibility that other cell types including low-density neutrophils may have contributed to these immunosuppressive responses. However, the expansion of the HLA-DR^−^ CD14^+^ population was directly associated with the reduction of ISGs and T and B cell pathways following the ALVAC + gp120 alum boosts.

Our data point to a complex interplay between the CD14^+^ and CD16^+^ monocyte subsets and MDSCs, via arginase activity and inflammasome activation. The arginase activity was inversely associated with the frequency of classical monocytes, with inflammasome activation, all correlates of decreased risk of SIV acquisition, and positively associated with the frequency of CD16^+^ monocytes. Together, these findings support the existence of a complex crosstalk between immune-activating and suppressive monocytic innate cells, in which the inflammasome activation and arginase catabolism of L-arginine are central components.

We have previously shown that classical monocytes were associated with protective Th2 cell responses (32). The levels of HLA-DR^−^CD14^+^ cells, arginase levels, and NO pathways all associated with decreased adaptive T and B immune responses, including SIV Gag-specific CD8^+^ T cells.

Our findings indicate a negative role for MDSCs in protection, however given the contradictory effects of immune suppressive cells in other viral infections (52, 58), it is tempting to speculate that MDSCs may have also contribute to protection from virus acquisition, for example by decreasing inflammation, thus reducing vulnerable HIV targets, such as activated CD4^+^ T cells. Results from HIV vaccine trials in humans and macaques suggest that inducing stronger adaptive immune responses may not be advantageous, as too much inflammation may increase HIV targets and thereby exacerbate infections (48, 59–62). Indeed, we found that the DNA-primed strategy induced fewer T cell responses and pro-inflammatory cytokines than both an Adenovirus-based vector (Ad26)-primed vaccine strategy and the MF59-adjuvanted vaccine, though the former did achieve superior protection (32, 49). In the current study, we did not observe any associations between specific CD4^+^ T cells and MDSCs, but this could perhaps be due to the time points chosen to collect blood samples.

Given the strong immunosuppressive capacity of MDSCs on CD4^+^ T helper cells, and the observed decrease in specific CD8^+^ T-cell responses observed also in our study, it is nevertheless possible that MDSCs might have affected vaccine-induced Th1- cell responses. We have previously identified Th1- cell responses to be harmful in ALVAC-vaccinated macaques (48, 49, 63), and limited induction of MDSC or MDSC-like cells may thus be partially beneficial in controlling inflammation and HIV target cells, particularly at mucosa sites.

**Figure 7:**
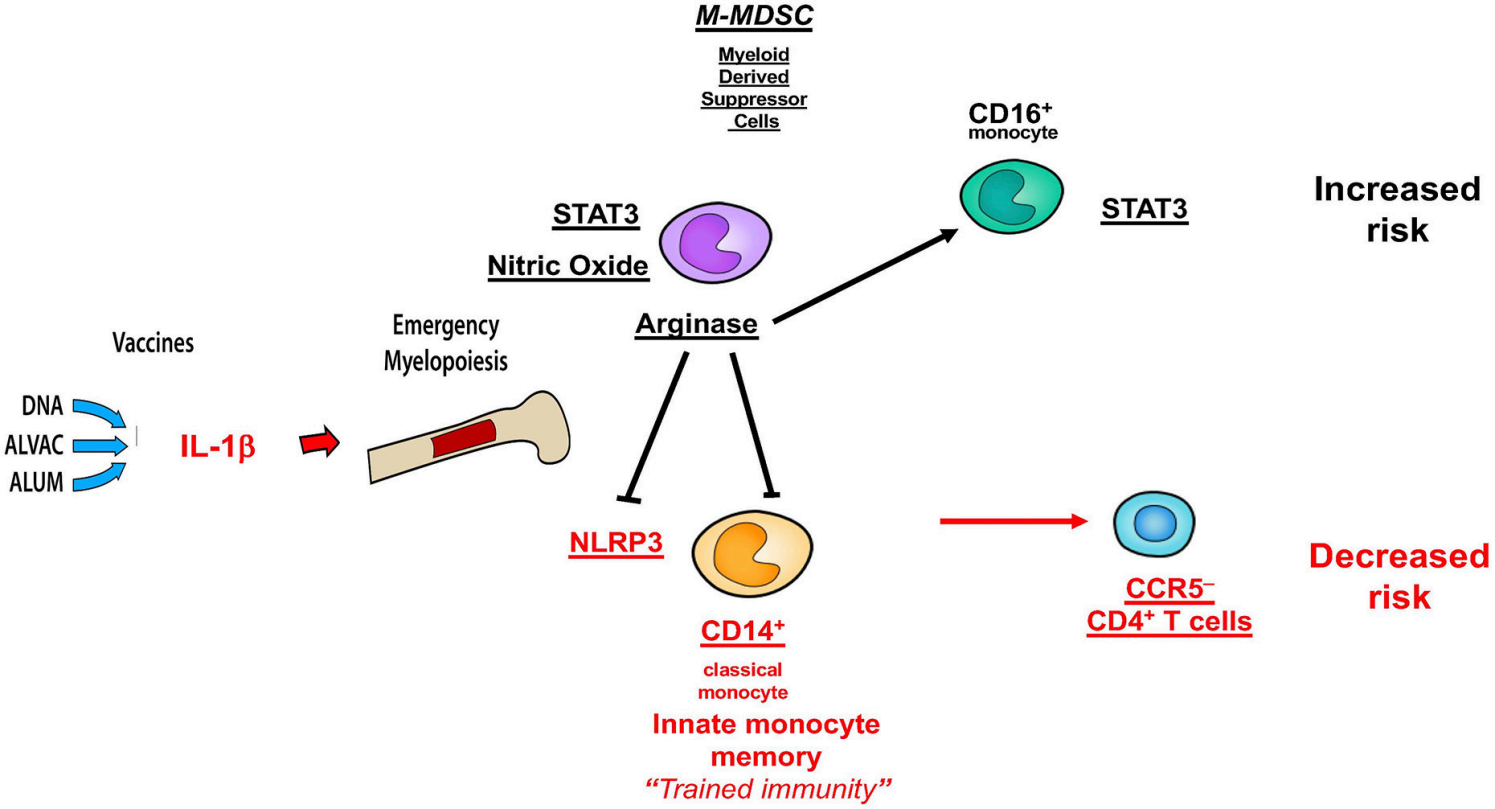
Schematic of correlates of decreased or increased risk of SIV acquisition in the DNA-SIV + ALVAC-SIV + gp120-alum strategy and their crosstalk. Cell subsets in black are associated with an increased acquisition risk, while those in red are associated with protection from SIV acquisition.

Collectively, the data presented here and those published in ref 32 demonstrate that MDSC and CD16^+^ monocytes have an opposite effect on the efficacy of the DNA + ALVAC + gp120 HIV vaccine candidate than innate classical CD14^+^ monocytes (Figure 7), underscoring the fundamental role of myeloid cells in shaping protective immune responses. A better understanding of the role of MDSCs in vaccine-mediate protection will be instrumental to improve the efficacy of HIV vaccine candidates, as well as vaccines against other human pathogens.

## Contribution to the Field Statement

A preventive vaccine for HIV is urgently needed. A vaccine using a Canarypox virus vector ALVAC was tested in a Thailand clinical (Thai) trial and, for the first time, resulted in significant protection from HIV acquisition. The level of protection afforded by this vaccine was limited, and this strategy must be improved. In the current study, we furthered our understanding of how this partial protective HIV vaccine candidate harnesses innate myeloid-derived cells and their role in vaccine efficacy. We show that immunosuppressive cells called MDSCs may interfere with the proper induction of T and B cell signals and specific CD8^+^ T cell responses, that are in turn needed to clear HIV infection. We also analyzed the immune suppressive mechanisms of MDSCs that are central to their harmful role. Altogether, our results underline the complexity of the immune system and suggest ways to strengthen the effectiveness of current HIV candidate vaccines.

### Code Availability

All source codes used are available https://github.com/sekalylab/mdsc.

## Data Availability

Microarray data can be obtained at the National Center for Biotechnology Information Gene Expression Omnibus (http://www.ncbi.nlm.nih.gov/geo) under accession number GEO: GSE108011.

## Ethics Statement

The study was conducted as previously described (32). All animals used in this study were colony-bred rhesus macaques (*Macaca mulatta*) provided by Covance Research Products. Monkeys were housed and handled in accordance with the standards of the Association for Assessment and Accreditation of Laboratory Animal Care International, and the care and use of the animals complied with all relevant institutional (U.S. National Institutes of Health) guidelines. The protocol (AUP 491) was approved by the Advanced BioScience Laboratories Institutional Animal Care and Use Committee.

## Author Contributions

GF designed the study and wrote the paper with MV, who also performed data analyses and prepared the figures. SF and R-PS analyzed the gene expression data, performed the correlates of risk analyses, prepared the figures, and helped write the manuscript. DB performed the flow cytometry for monocytes in blood and some correlative analyses. KF, MR, and RK performed the intracellular cytokine analysis. IS and MB performed the ELISA and Luminex assays. JB and YS provided suggestions for the identification of MDSCs by FACs. All the authors performed critical review of the manuscript.

### Conflict of Interest Statement

The authors declare that the research was conducted in the absence of any commercial or financial relationships that could be construed as a potential conflict of interest. The handling Editor declared a past co-authorship with one of the authors R-PS.
