# Supplementary material for: Myeloid Cell Crosstalk Regulates the Efficacy of the DNA/ALVAC/gp120 HIV Vaccine Candidate
Source: Front Immunol. 2019 May 14;10:1072. doi: 10.3389/fimmu.2019.01072 (PMC6527580; doi:10.3389/fimmu.2019.01072)
Supplement: Supplementary file 2 [file Data_Sheet_1.PDF]

Supplementary Material

1 Supplementary Data

1.1 Supplementary Figures

A B

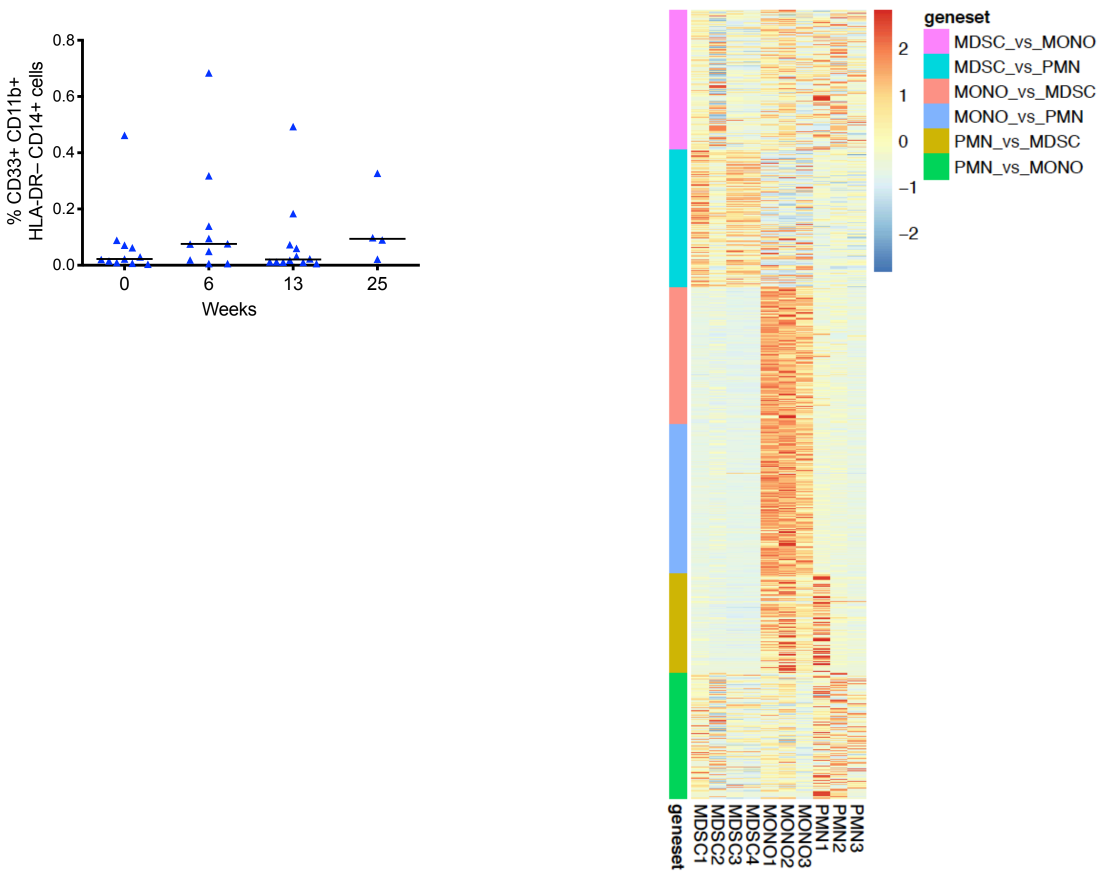

Supplementary Figure 1. (**A**) Heatmap showing the expression of transcriptomic markers of MDSCs (cyan) in sorted MDSCs, monocytes, and neutrophils of the Heim *et al.* dataset. The top 200 genes induced in Ly6G^{+} Ly6C^{+} CD11b^{high} MDSCs compared to sorted Ly6G^{+}Ly6C^{+}CD11b^{low} neutrophils (fold-change > 1 and likelihood-ratio test $P < 0.05$, ranked by increasing P) identified by Heim *et al.* were considered transcriptomic markers of MDSCs.

1.2 Supplementary Tables

Timepoint	NES	NOM p-val	FDR q-val	LEADING_EDGE
1st ALVAC + 24hours	2.06	0	0	MS4A6A,TNFRSF1A,ENTPD1,TIPARP,LPCAT2,MS4A4A,NOD2,DGAT1,NRG1,TREM1,PTGS2,CCNJL,PARP10,PARP9,ANTXR2,IFIT1,OASL,TNRC18,SAMSN1,CWC25,CSF2RB,ETS2,VPS37B,THBS1
1st ALVAC + 24hours	1.43	0.0251	0.0297	VAT1,SCARB2,ATXN1,VAV1,RNF13,IGSF6,MAG,FBXO38,ZNFX1,MFAP5,GSK3B,VCL,AATK,MCTP1,LRG1,ZBTB9,ASTN2,RHBDF2,MAPK8IP3
2nd ALVAC + 1week	1.65	0.00493	0.0134	PGD,FBXL20,EVI2B,HERC1,PHF20L1,AP3S1,FNBP1L,WBP4,SSH2,AKAP13,ACRBP,DUSP13,ZNF768,OTUD7A,EPS15,CLIC1,HMGCL,ILKAP,TERF2IP,MACROD2,ADCYAP1R1,CTCFL,CDH26,KRBA1,JMY,AGL,NFIA
2nd ALVAC + 24hours	2.27	0	0	MS4A6A,TIPARP,LPCAT2,NOD2,ENTPD1,TNFRSF1A,SAMSN1,PARP9,CSF2RB,TREM1,MS4A4A,IFIT1,PTGS2,OASL,NRG1,CCNJL,DGAT1,PARP10,ELL2,ANTXR2,ETS2,TNRC18,CWC25,THBS1,SOCS1,PLEKHG6,ADAM9
2nd ALVAC + 24hours	1.39	0.0269	0.0462	SCARB2,RNF13,ATXN1,VAV1,IGSF6,FBXO38,VAT1,MAG,ARNTL2,ZNFX1,VCL,MFAP5,AATK,MCTP1

Supplementary Table 1. Gene set enrichment analysis of MDSCs, monocytes, and neutrophil markers among genes differentially expressed post-vaccination. *Timepoint* corresponds to the timepoint at which the gene-expression was measured. *NES* refers to the normalized enrichment score. *NOM p-val* and *FDR q-val* respectively correspond to the nominal *P* value and the false-discovery of pathway enrichment. *LEADING_EDGES* are the genes contributing to pathway enrichment.

Timepoint	NES	NOM p-val	FDR q-val	LEADING_EDGE
1st ALVAC + 24hours	1.67	0	0.0114	TTC37,SNX19,COPS3,VIM,IMMP1L,CLNS1A,PGM3,SMPDL3A,EBAG9,PARS2,RCN2,WDR70,AP1B1,EXOSC7,IARS2,PLK4,SLC12A9,E2F1,LARP7,SMC3,RAD54L,RTN1,TASP1,KLHDC1,KBTBD11,ARL4C,DHX57,TSHZ1,CEP55,ADAM15,PATZ1,INPP4B,PLEKHH3,GPRIN3,ACAA1
1st ALVAC + 2weeks	-1.45	0.0214	0.0453	CD33,AMBP,TNRC18,PARP10,SKIL,TSPAN13,ECM1,SOCS1,MS4A6A,THBS1,MARCH7,NR4A3,ALDH18A1,STON2,PTGS2,DGAT1,NOD2,CHST11,ANTXR2,CCNJL,TREM1,TNFRSF1A,VPS37B
1st ALVAC + 2weeks	1.70	0	0.00432	INPP4B,CLNS1A,PSMF1,SYTL2,LARP7,ARL4C,TASP1,EBAG9,EIF3M,IARS2,RPS6KB2,TSHZ1,MCOLN2,TTC37,E2F8,PLK4,RAD54L,UGGT1,ENOX2,MAP3K10,NUDCD1,COPS3,EXOSC7,PRMT7,KIF22,RCN2,FOXM1,CORO2A,LHPP
1st ALVAC + 2weeks	-1.78	0	0.00207	DNASE1L3,MFAP5,BTBD9,ATP2B1,RNF44,CCDC157,GSK3B,SCARB2,RNF13,ZNFX1,XPC,MCTP1,VAV1,MAG,GIGYF2,IGSF6,VCL,ARNTL2,NCAM1,PLA2G7
2nd ALVAC + 24hours	1.81	0.00251	0.00126	LPCAT2,ELL2,MARCH7,NOD2,TIPARP,CSF2RB,SAMSN1,GMPR2,ENTPD1,CHD7,PLEKHG6,ADAM9,CASP8,CXCR5,IFIT1,DGAT1,MS4A4A,OASL,PARP9,THBS1,MS4A6A,MEIG1,SOCS1,ACTG1,PARP10
2nd ALVAC + 24hours	1.49	0.00458	0.0493	IMMP1L,CLNS1A,CEP55,COPS3,ENOX2,KBTBD11,EXOSC7,TASP1,RCN2,PLK4,SMPDL3A,TSPYL2,UFM1,BYSL,SNX19,EML4,E2F1,MAP3K10,ARL4C,VIM,CLUAP1,CORO2A,SMC3,E2F8,PARS2,RTN1,EFTUD2,TOP2A,UGGT1,INPP4B,PLXNA1,ADAM15

Supplementary Table 2. Gene set enrichment analysis of MDSCs, monocytes, and neutrophil markers among genes correlated with challenge. *Timepoint* corresponds to the timepoint at which the gene-expression was measured. *NES* refers to the normalized enrichment score. *NOM p-val* and *FDR q-val* respectively correspond to the nominal *P* value and the false-discovery of pathway enrichment. *LEADING_EDGES* are the genes contributing to pathway enrichment.

Supplementary Table 3. Gene set enrichment analysis of pathways enriched among genes correlated with HLA-DR^{-}CD14^{+} and challenge. *Timepoint* corresponds to the timepoint at which the gene-expression was measured. *Name* refers to the MSigDB gene set name. *NES* refers to the normalized enrichment score. *NOM p-val* and *FDR q-val* respectively correspond to the nominal *P* value and the false-discovery of pathway enrichment. *LEADING_EDGES* are the genes contributing to pathway enrichment(*in Excel format*).

Supplementary Table 4. Levels of ROS/RN and Kynurenine (Kyn)/Tryptophan(Tryp) ratio in plasma at week 6 and 25. nd = not determined.

	**Kyn/Tryp**	**Kyn/Tryp**	**ROS/RN**	**ROS/RN**
Animal	Week 6	Week 25	Week 6	week 25
P218	0.116752	0.08	nd	510.7599
P222	0.098118	0.07	nd	468.2685
P226	nd	nd	419.2767	505.7311
P231	0.049471	0.04	449.5372	442.725
P235	nd	nd	439.8207	521.6069
P239	0.071039	0.09	392.9048	644.364
P255	0.078182	0.08	414.5793	538.1454
P257	nd	nd	453.8741	nd
P261	0.064709	0.04	486.532	501.2383
P268	0.099919	0.06	376.4053	466.602
P273	0.092488	0.08	414.5793	680.3258
P278	nd	nd	477.6147	598.5104
